# Wistar-Kyoto Female Rats Are More Susceptible to Develop Sugar Binging: A Comparison with Wistar Rats

**DOI:** 10.3389/fnut.2017.00015

**Published:** 2017-05-09

**Authors:** Helena Papacostas-Quintanilla, Víctor Manuel Ortiz-Ortega, Carolina López-Rubalcava

**Affiliations:** ^1^Laboratorio de Psicofarmacología y Trastornos de la Alimentación, Departamento de Farmacobiología, CINVESTAV, Ciudad de México, Mexico; ^2^Departamento de Fisiología de la Nutrición, Instituto Nacional de Ciencias Médicas y Nutrición Salvador Zubirán, Ciudad de México, Mexico

**Keywords:** Wistar-Kyoto, binge eating, strain, animal model, eating disorders, sex differences

## Abstract

The hedonic component of the feeding behavior involves the mesolimbic reward system and resembles addictions. Nowadays, the excessive consumption of sucrose is considered addictive. The Wistar-Kyoto (WKY) rat strain is prone to develop anxiety and addiction-like behavior; nevertheless, a lack of information regarding their vulnerability to develop sugar binging-like behavior (SBLB) and how it affects the reward system persist. Therefore, the first aim of the present study was to compare the different predisposition of two rat strains, Wistar (W) and WKY to develop the SBLB in female and male rats. Also, we studied if the SBLB-inducing protocol produces changes in anxiety-like behavior using the plus-maze test (PMT) and, analyzed serotonin (5-HT) and noradrenaline (NA) concentrations in brain areas related to anxiety and ingestive behavior (brain stem, hypothalamus, nucleus accumbens, and amygdala). Finally, we evaluated whether fluoxetine, a drug that has been effective in reducing the binge-eating frequency, body weight, and severity of binge eating disorder, could also block this behavior. Briefly, WKY and W female rats were exposed to 30% sucrose solution (2 h, 3 days/week for 4 weeks), and fed up *ad libitum*. PMT was performed between the last two test periods. Immediately after the last test where sucrose access was available, rats were decapitated and brain areas extracted for high-performance liquid chromatography analysis. The results showed that both W and WKY female and male rats developed the SBLB. WKY rats consumed more calories and ingested a bigger amount of sucrose solution than their W counterpart. This behavior was reversed by using fluoxetine, rats exposed to the SBLB-inducing protocol presented a rebound effect during the washout period. On female rats, the SBLB-inducing protocol induced changes in NA concentrations on WKY, but not on W rats. No changes were found in 5-HT levels. Finally, animals that developed SBLB showed increased anxiety-like behavior in the PMT. In conclusion, WKY female rats can be considered as a more susceptible rat strain to develop SBLB.

## Introduction

The binge eating disorder (BED) was defined for the first time as a specific eating disorder in the Diagnostic and Statistical Manual of Mental Disorders Fifth Edition (DSM-V). This disorder is characterized by recurrent binge eating episodes (eating large amounts of food in a short period with an associated sense of loss of control, at least once a week for 3 months) without any inappropriate compensatory behaviors. Palatable food (high in fat, simple carbohydrates, or a mix of both) is commonly consumed during these binge eating episodes (1–3). Today BED is the most prevalent eating disorder worldwide, being more common in women than men (2–2.5 women per men) (4–6) and with a highly heritable phenotype (7). A high number of patients diagnosed with this disorder are also overweight or obese, being the excess body fat one of the key risk factors for the development of non-communicable diseases (6, 8, 9).

Animal models have been used to understand the neurobiology of BED; these models try to resemble the characteristics of the binge eating episode in humans. However, the associated loss of control over eating, central to the definition of a binge eating episode is difficult to measure because it is an intrinsic human subjective experience, thus limiting its interpretation (3, 10). Nevertheless, animal models represent a fair enough approach that has significantly contributed to the understanding of possible causes or consequences of binge eating behavior.

A wide range of palatable food and intake schedules have been used to induce binge eating-like behavior in animal models. Because both, high-sugar and high-fat foods are related to binge eating, different research groups have chosen to use sucrose solution (10–30%) to induce it. This solution is either used on a daily basis or three times per week and for long or short periods of time (11–14); in these cases, specifically studying the SBLB (15–20).

There is a high comorbidity between eating disorders (ED) and anxiety-related disorders. The Wistar-Kyoto (WKY) rat strain, developed as a control animal for the spontaneous hypertensive rat, is recognized to be more susceptible to develop anxiety and depression-like behavior (21, 22). Moreover, binge eating has been compared with addictive behaviors (23–27). Studies have shown that the WKY rat strain has different reward-related responses and seeking behavior when exposed to cocaine and alcohol (28), but there is no information about possible differences in their feeding behavior and vulnerability to develop sugar binging-like behavior (SBLB) (29). Therefore, the first aim of this research was to analyze whether the WKY rats, due to their natural anxiety and depression related profile, had a higher predisposition to develop SBLB in comparison with the Wistar (W) rat strain. Also, because the prevalence in humans is greater in women than men, we analyzed whether there were sex differences in the development of SBLB. Moreover, to analyze if sugar binge increases anxiety-like behavior, animals that showed SBLB were subjected to an animal model of anxiety, the plus-maze test (PMT).

Some neurotransmitters like noradrenaline (NA) and serotonin (5-HT), known to be involved in the regulation of these psychiatric disorders, also play a role in the modulation of feeding behavior (30–33). Thus, to further characterize the consequences of SBLB, we also studied NA and 5-HT concentrations in the brain stem, amygdala, hypothalamus, and nucleus accumbens (NAcc); brain areas related to the regulation of both, feeding and anxiety behaviors.

Finally, some 5-HT reuptake inhibitors have been suggested for the treatment for BED (29). Fluoxetine, a selective serotonin reuptake inhibitor, is sometimes used to treat ED in humans and has been effective in reducing binge-eating frequency, body weight, and overall severity of BED (34–36). Therefore, another aim of the present study was to analyze if this drug was also able to reverse SBLB. Because previous studies have shown that WKY rats have a blunted response to serotonergic compounds (37, 38), this experiment was performed only on W female rats.

Our hypothesis was that the female WKY rats would show increased SBLB and anxiety-like behavior, which could correlate with changes in the concentrations of NA and 5-HT in brain areas related to the regulation of feeding and anxiety.

## Materials and Methods

### Animals

We used 8-week-old female and male W and WKY rats, provided by our breeding facilities. Rats were housed five per cage with *ad libitum* access to non-palatable food and water. Animals were kept in a controlled environment (22 ± 2°C) and inverted light schedule (12 h/12 h light/dark cycle). All experimental procedures were approved by CINVESTAV’s ethic committee (Protocol 0179-16) and followed the regulations established by the Mexican Official Norm (NOM-062-ZOO-1999) for the use and care of laboratory animals.

### Methods

#### SBLB Induction Protocol

During the first week of the protocol, and to avoid handling and separation related stress, on Monday, Wednesday, and Friday, animals of all groups were placed in individual cages for 2 h.

Afterward, from week 2 to 5 on “test days” (Monday, Wednesday, and Friday), 2 h after the beginning of the dark cycle, animals were separated into individual cages for 2 h. During this period, two sipper tubes were available, one with sucrose solution at 30% and other with water (control groups had two sipper tubes with water) and food *ad libitum*. After this period, animals were brought back to their group home cage were water, and non-palatable food (LabRat 5008^®^) were always available. Control group followed the same protocol without having access to the sucrose solution during isolation periods.

According to Perello et al. (10), a binge eating episode was considered as such when animals consumed significantly more calories than the control group, and in the present study, we also considered as a binge parameter when animals exceed their total gastric capacity (see below).

Binge eating disorder patients usually try to compensate the high caloric intake during binge eating episodes by going through short dieting periods (39). In order to analyze if the rats followed a similar pattern, the daily total caloric intake (of palatable and non-palatable food) was measured when separated individually and averaged out when at their home cages. In this analysis, if daily caloric consumption of animals with SBLB is similar to the control group it means that they were able to compensate the high caloric intake during test periods through a reduction of their intake while in home cages.

#### Calculation of Gastric Capacity

One of the characteristics of a binge eating episode is to eat until feeling uncomfortably full (DSM-V), which is related to an excessive distention of the stomach. Food has different caloric densities (calories per square centimeter) (40). Thus, comparing the size of the stomach vs. the volume of food consumed can give us an idea of the stomach distention regardless of caloric consumption. To calculate the gastric capacity we used the formula created by Bull and Pitts (41). This formula states that the relationship between body weight (*X*, in grams) and stomach capacity (*Y*, in milliliters) for rats was: *Y* = 0.038*X* − 0.770 (41).

#### Plus-Maze Test

As for the analysis of anxiety-like behavior, animals were subjected to the PMT between test day 11 and 12. This test is based on the animal’s natural aversion to open spaces. The PMT consisted of an acrylic maze with four arms (50 cm × 10 cm), two open and two closed. The maze was elevated 30 cm from the ground. Two hours after the beginning of the dark period, animals were placed in the PMT for 5 min. All tests were recorded, and videos were analyzed by a trained and experienced observer unaware of the treatments. We measured the following parameters: (a) the total number of rearings; (b) time in open and closed arms; (c) total entries to close and open arms; and (d) total time spent immobile (freezing behavior). A decrease in both, time spent in the open arms and total number of entries to the open arms, are considered as an increase in anxiety-like behavior (42).

#### Analysis of Total Tissular Concentration of NA or 5-HT of Specific Brain Areas

We used high-performance liquid chromatography (HPLC) to analyze NA and 5-HT concentrations in different brain areas. Brain samples were extracted over ice and placed in an antioxidant solution (acetic acid 0.1 M, ascorbic acid 0.5 mM, and EDTA 0.27 mM). Immediately after, the samples were homogenized and centrifuged at 14,000 rpm at 4°C for 20 min. Hundred microliters of supernatant were first filtered with a nylon filter (Millex, USA: REF: SKHNX13NK) and then injected into the HPLC.

The mobile phase (0.98 mM sodium octyl sulfate, 0.43 mM EDTA, 33.33 mM NaH_2_PO_4_, 10 mM NaCl, methanol 130 ml, pH 4.0) was pumped through a reverse-phase 3 µm catecholamine column [Grace Davison Discovery Science (SN: 610080721), IL, USA] with a pre-column with the same characteristics at a flow rate of 1 ml/min, with an oxidation potential of 800 mV, sensibility of 5 nA and controlled temperature of 29°C.

#### Sucrose Preference Test

Male and female WKY and W rats (*n* = 8 per group) were singly housed during 48 h in individual cages with continuous access to two bottles, one with 30% sucrose solution and the other with water; the food was available *ad libitum*. Daily ingestion of sucrose solution, water, and chow was measured.

### Experimental Design

#### Differences between Sex and Strain in Response to the SBLB Protocol and Development of Anxiety-Like Behavior

**Figure 1:**
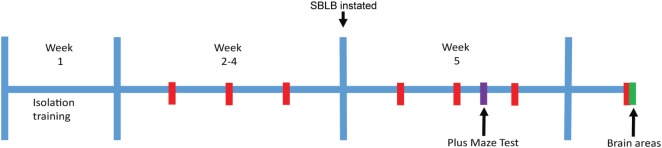
**Stressor-free binge eating-like behavior inducing protocol**. Red: 2 h test periods where sucrose solution access is granted. Purple: plus-maze test. Green: sacrifice for tissue extraction.

In order to evaluate whether sex and strain differences play a role in the vulnerability to develop SBLB, the response to the SBLB-inducing protocol was assessed in four groups (female and male W and WKY rats, 10–12 animals per group). To compare the caloric intake between strains and sex we normalized the results using weight, all results are shown as kilocalories per gram of weight or milliliters per gram of weight. Between the 11th test day and 12th test day, we subjected the animals to the PMT (see Figure 1) to evaluate anxiety-like behavior.

#### Analysis of NA and 5-HT Total Tissular Concentrations in Brain Areas Related to Anxiety and Feeding Behavior

Animals used for the behavioral analyses were decapitated immediately after the last 2 h test period (on the 12th test). We removed the brains and dissected them over ice obtaining the following areas: brain stem, hypothalamus, amygdala, and NAcc. Dissected areas were weighted and kept at −80°C in Eppendorf tubes with 300 µl of antioxidant solution until HPLC analysis.

#### Analysis of the Effect of Fluoxetine on SBLB

To analyze if fluoxetine could block SBLB, we subjected female W rats (10 per group) to the SBLB protocol; once it was established, we tested a subchronic treatment of fluoxetine (10 mg/kg i.p. for 5 days, 30 min before being isolated for sugar intake). Control animals were administered with vehicle (saline solution 0.9%). Food intake and weight were registered the full duration of the experiment and continued 1 week after the fluoxetine treatment concluded (washout period).

### Statistical Analysis

All statistical analysis were made using Sigma Plot 12.0, and the graphs were made using GraphPad Prism 6.0. One-, two- and three-way ANOVAs were used depending on the number of variables studied. When comparing data to an initial value, repeated measure ANOVA was used. All results are shown as mean ± SEM.

## Results

### Differences between Sex and Strain in Response to the SBLB Protocol

**Figure 2:**
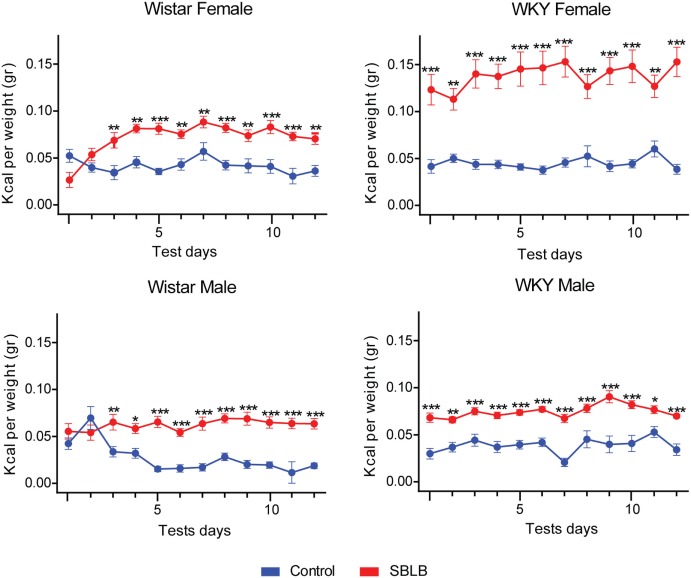
**The sugar binging-like behavior-inducing protocol was successful in all groups: Wistar female and male rats (left panels) developed binge eating episodes after two sucrose access periods while Wistar-Kyoto (WKY) females and male started binge eating from the beginning (right panels)**. Two-way ANOVA with Sidak’s multiple comparison test per panel, *n* = 10–15 animals per group (*P < 0.05, **P < 0.01, ***P < 0.001).

Figure 2 shows differences between the total caloric intake of SBLB vs. control during the test period in WKY and W rats. The SBLB-inducing protocol produced binge episodes in all studied groups. In W female and male rats, these episodes were seen after the second-test period, while in WKY female and male rats, started in the first test period. When analyzing the area under the curve during test periods, W SBLB female rats ingested 172% of the calories consumed by their control group, W SBLB male rats, 207%, WKY SBLB female rats, 321%, and finally the WKY SBLB male rats, 193% (data not shown).

**Figure 3:**
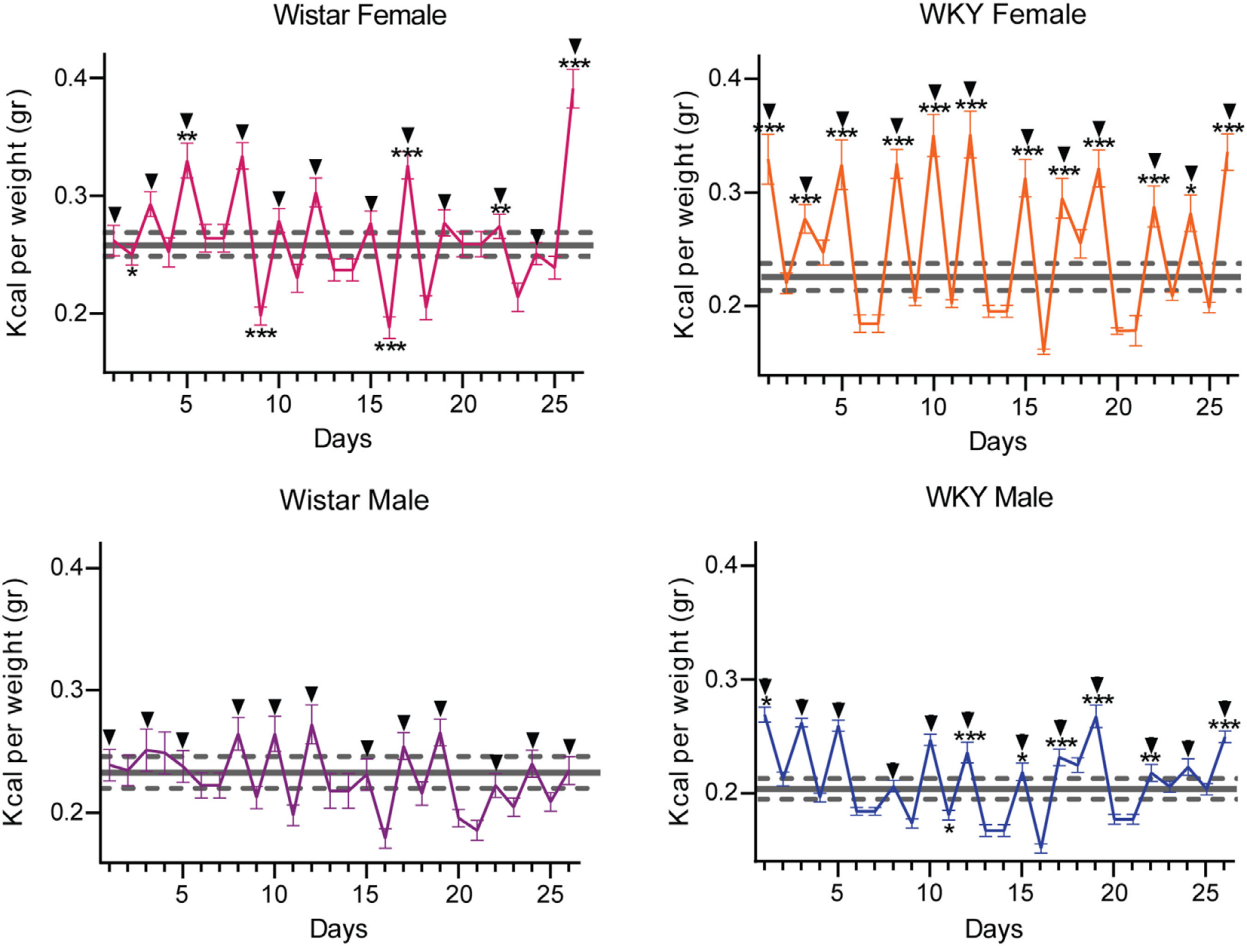
**Caloric consumption variation during test and no test days during the sugar binging-like behavior protocol**. The data of the control group are represented as mean ± SEM (gray doted lines); test days distinguished by a black arrow on top. Two-way ANOVA using variation factors time and treatment with Sidak’s multiple comparisons test. (ANOVA was calculated using the raw data.) (*P < 0.05, **P < 0.01, ***P < 0.001).

Figure 3 shows animals ability to compensate daily caloric intake unbalanced by the high caloric intake during test periods. It is observed that SBLB rats during test days increase their caloric intake in comparison to the control but compensate with the intake on home cages on no test days leading to a cycling intake behavior.

This behavior became significantly different when comparing it with the control group in three of our experimental groups. In female W rats, we found four significantly higher test days (upper left panel), in the WKY male rats 7 days (lower right panel) and in the WKY female rats, 12 days (upper right panel). Male W rats only showed a tendency to develop the behavior, never developing a statistically different intake on test days (lower left panel).

When averaging the total caloric intake of test and no test days (see Table 1), the WKY female rats were the only SBLB group that showed a significant increase in this parameter (*P* < 0.01).

**Table 1:** **Average of total caloric consumption (test days + no-test days) normalized by weight during the sugar binging-like behavior (SBLB) protocol**.

Strain	Sex	Treatment	Mean ± SEM	*N*	*T*	*P*
Wistar	Female	Control	0.2710 ± 0.0100	10	0.5880	0.5592
		SBLB	0.2651 ± 0.0177	10		
	Male	Control	0.2276 ± 0.0124	9	0.1272	0.8993
		SBLB	0.2284 ± 0.0132	10		

Wistar-Kyoto	Female	Control	0.2188 ± 0.0109	12	2.601	0.0122**
		SBLB	0.2539 ± 0.0266	12		
	Male	Control	0.2006 ± 0.0089	10	1.296	0.2011
		SBLB	0.2113 ± 0.0120	10		

Unpaired T-test, **p < 0.01.

In order to analyze which variation factors (sex, strain, or treatment) were the ones influencing the changes in the mean caloric intake of the four different SBLB groups, we used a three-way ANOVA test. In this analysis, we found that all the variation factors studied caused statistically significant changes in the mean caloric intake (sex *P* < 0.001, strain *P* < 0.001, and treatment *P* < 0.05), same as the interaction between the strain and the treatment (*P* < 0.05) (see Table 2).

**Table 2:** **Three-way ANOVA of the mean caloric intake (test days + no-test days) normalized by weight during the sugar binging-like behavior protocol using as variation factors the strain, sex, and treatment**.

Variation factor	DF	SS	MS	*F*	*P*
Strain	1	0.0378	0.0378	29.672	<0.001
Sex	1	0.0643	0.0643	50.491	<0.001
Treatment	1	0.00543	0.00543	4.266	0.04
Strain × sex	1	0.00123	0.00123	0.97	0.326
Strain × treatment	1	0.00847	0.00847	6.65	0.011
Sex × treatment	1	0.00105	0.00105	0.825	0.365
Strain × sex × treatment	1	0.00320	0.00320	2.509	0.115

**Figure 4:**
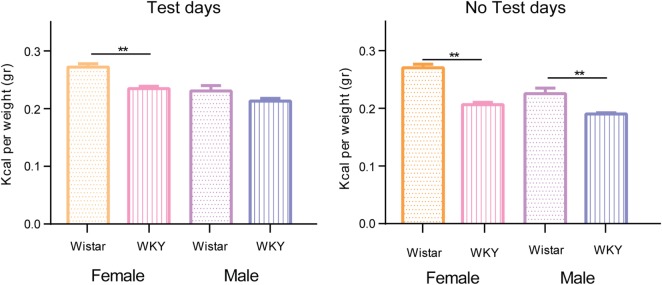
**Comparison of the mean caloric consumption of control groups separated by test and no test days**. Two-way ANOVA using variation factors time and treatment with Sidak’s multiple comparisons test (**P < 0.01).

Figure 4 shows the comparison between the mean caloric intake between sex and strain on test and no test days of the control groups. On test and no test days, WKY rat strain consumes significantly fewer calories than the W rat strain, with the only difference of the male W rats on test days.

**Figure 5:**
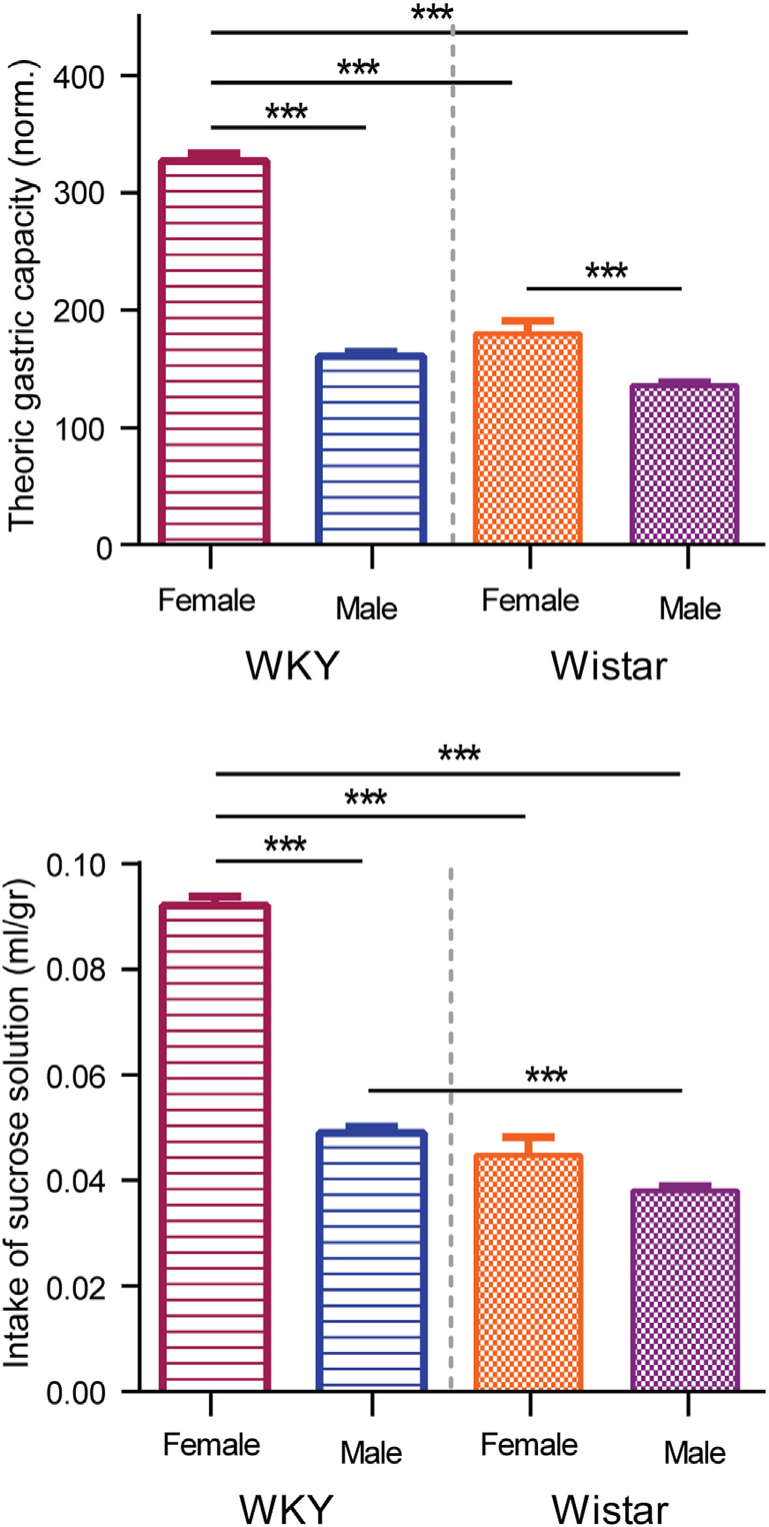
**Upper panel: comparison of milliliters of sucrose solution consumed during test periods**. Two-way ANOVA with Sidak’s multiple comparison test (***P < 0.001). Lower panel: comparison of the volume of food consumed normalized by the theoric gastric capacity. Two-way ANOVA with Sidak’s multiple comparison test (***P < 0.001).

Figure 5 shows the average of sucrose intake during the SBLB protocol (lower panel) and the average volume of food consumed during the test period (upper panel, percentage of theoric gastric capacity). The WKY female rats consumed significantly more milliliters per gram of weight of sucrose solution than the other three SBLB groups (206% vs. W female, 242% vs. W male, and 188% vs. WKY male, *P* < 0.001). Also, the WKY male rats consumed more sucrose solution than their W counterpart (129%, *P* < 0.001).

During test periods, on average the WKY female rats significantly exceeded their theoric gastric capacity when compared to the rest of the groups (203% vs. males WKY, 182% vs. females W, and 241% vs. males W, all *P* < 0.001); exceeding their gastric capacity more than three times (327% average). The W female rats consumed a bigger volume of food during test periods than the male W (133%).

**Figure 6:**
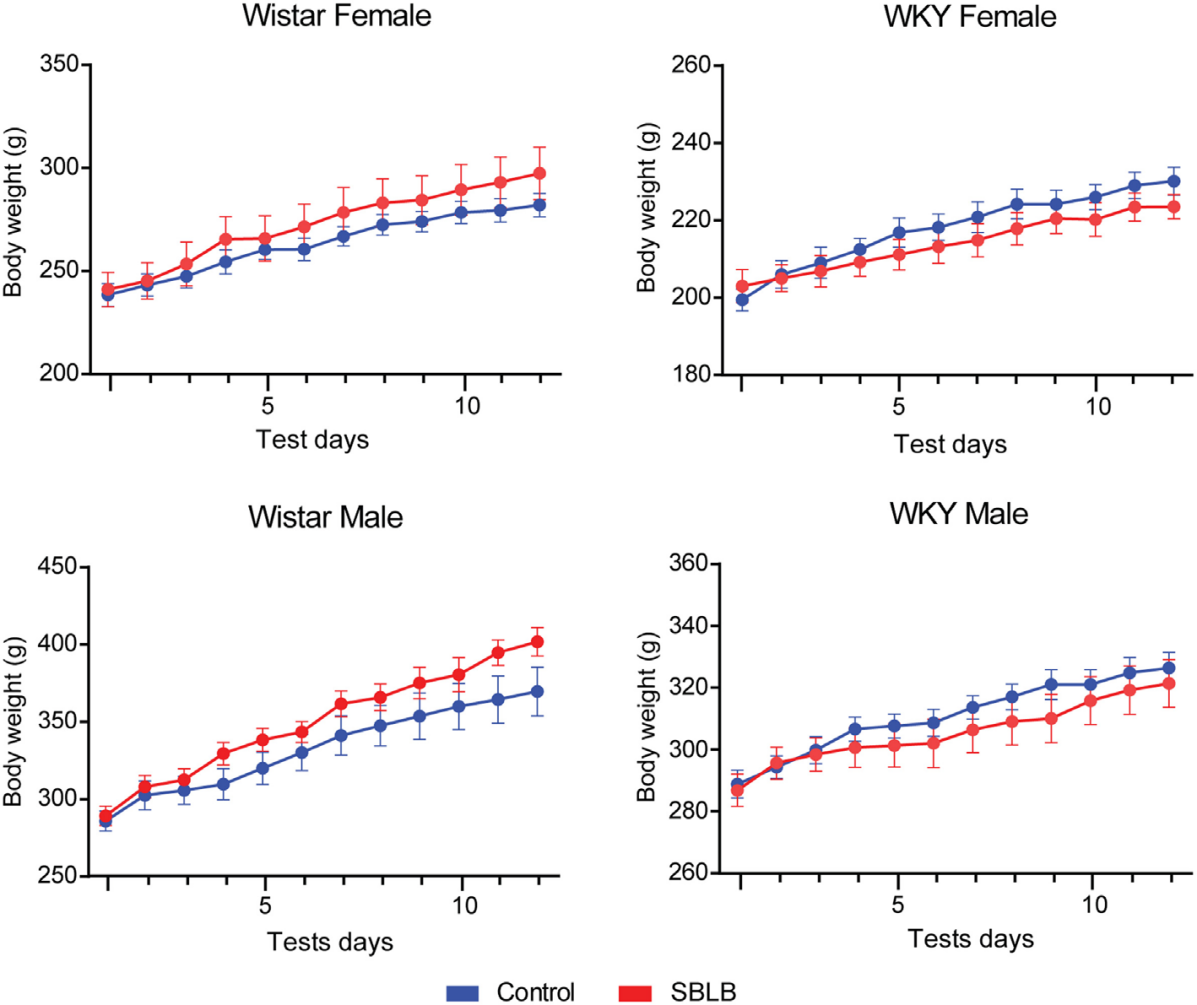
**Weight during the sugar binging-like behavior-inducing protocol**. One-way ANOVA, no significant differences found between treatments.

Finally, we did not observe differences in the growth curve between groups during the SBLB-inducing protocol (Table 3; Figure 6).

**Table 3:** **Weight change during the sugar binging-like behavior (SBLB) protocol, comparison between SBLB vs. control group per sex and strain**.

Strain	Sex	Treatment	ΔWeight (mean ± SEM)	*N*	*T*	*P*
Wistar	Female	SBLB	56.400 ± 6.309	10	0.0605	0.12
		Control	43.600 ± 4.693	10		
	Male	SBLB	102.889 ± 6.533	9	1.396	0.18
		Control	89.333 ± 4.721	10		

Wistar-Kyoto	Female	SBLB	22.833 ± 2.614	12	0.05376	0.95
		Control	22.545 ± 4.614	12		
	Male	SBLB	34.600 ± 5.207	10	0.3601	0.72
		Control	37.600 ± 6.503	10		

*Unpaired T-test*.

### Sucrose Preference Test

There were no differences between groups in sucrose solution intake during the 48 h sucrose preference test [W female 24.19 ± 1.397, WKY female 25 ± 2.44, W male 25.44 ± 2.031, WKY male 29.47 ± 2.663, one-way ANOVA, *F*(3, 58) = 1.165].

### Anxiety-Like Behavior Development As a Result of the SBLB Protocol in WKY and W Rats

**Figure 7:**
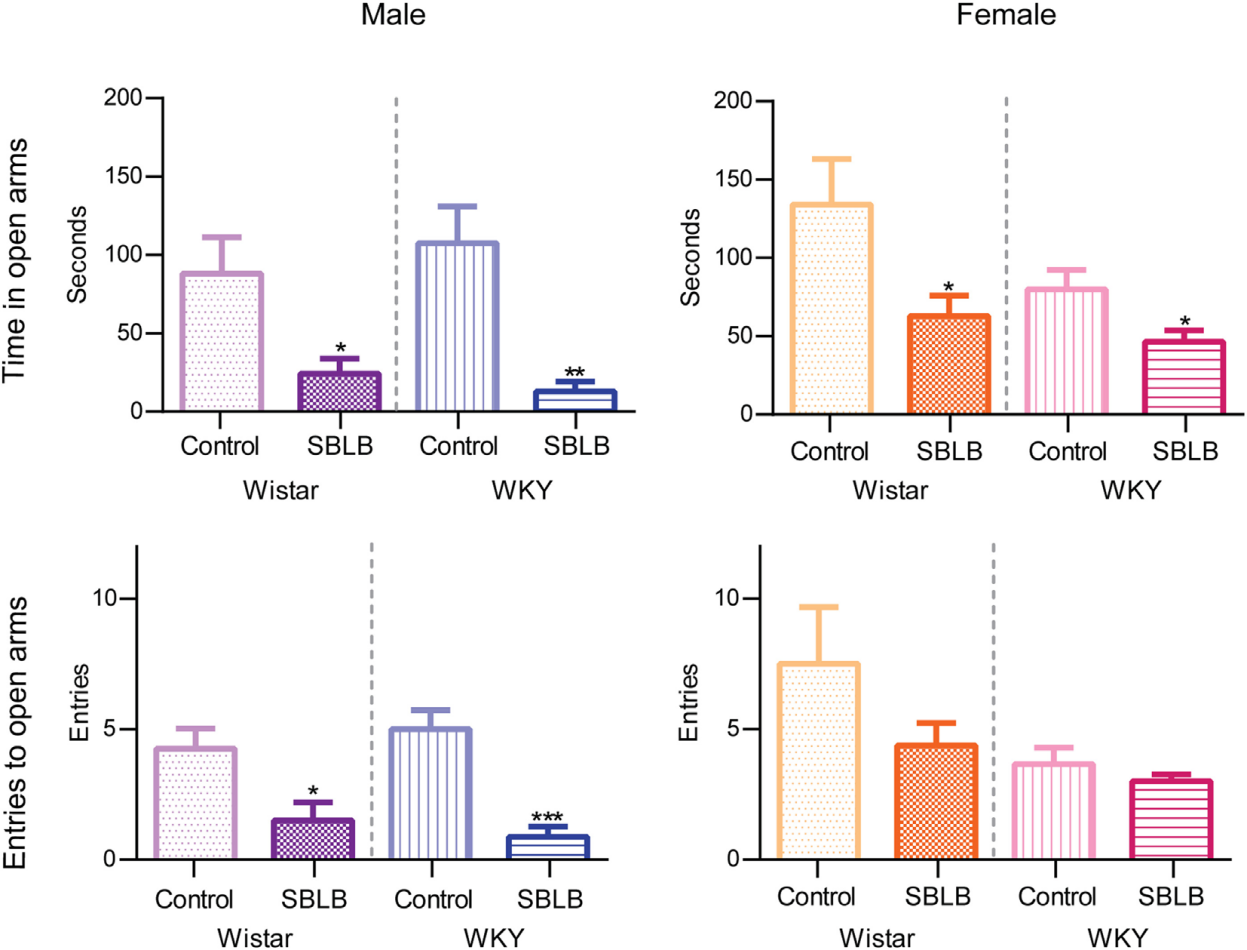
**Time spent in open arms and entries to open arms divided by sex and strain**. Two-way ANOVA with Sidak’s multiple comparison test (*P < 0.05, **P < 0.01, ***P < 0.001).

All SBLB groups compared to its control significantly reduced the time spent on the open arms of the maze (W female and WKY female *P* < 0.05, and W male and WKY male *P* < 0.01) (Figure 6, upper panels). Also, the female SBLB rats of both strains showed a tendency to decrease the number of entries to open arms. This tendency became statistically significantly on W and WKY male rats (Figure 7, lower panels).

**Figure 8:**
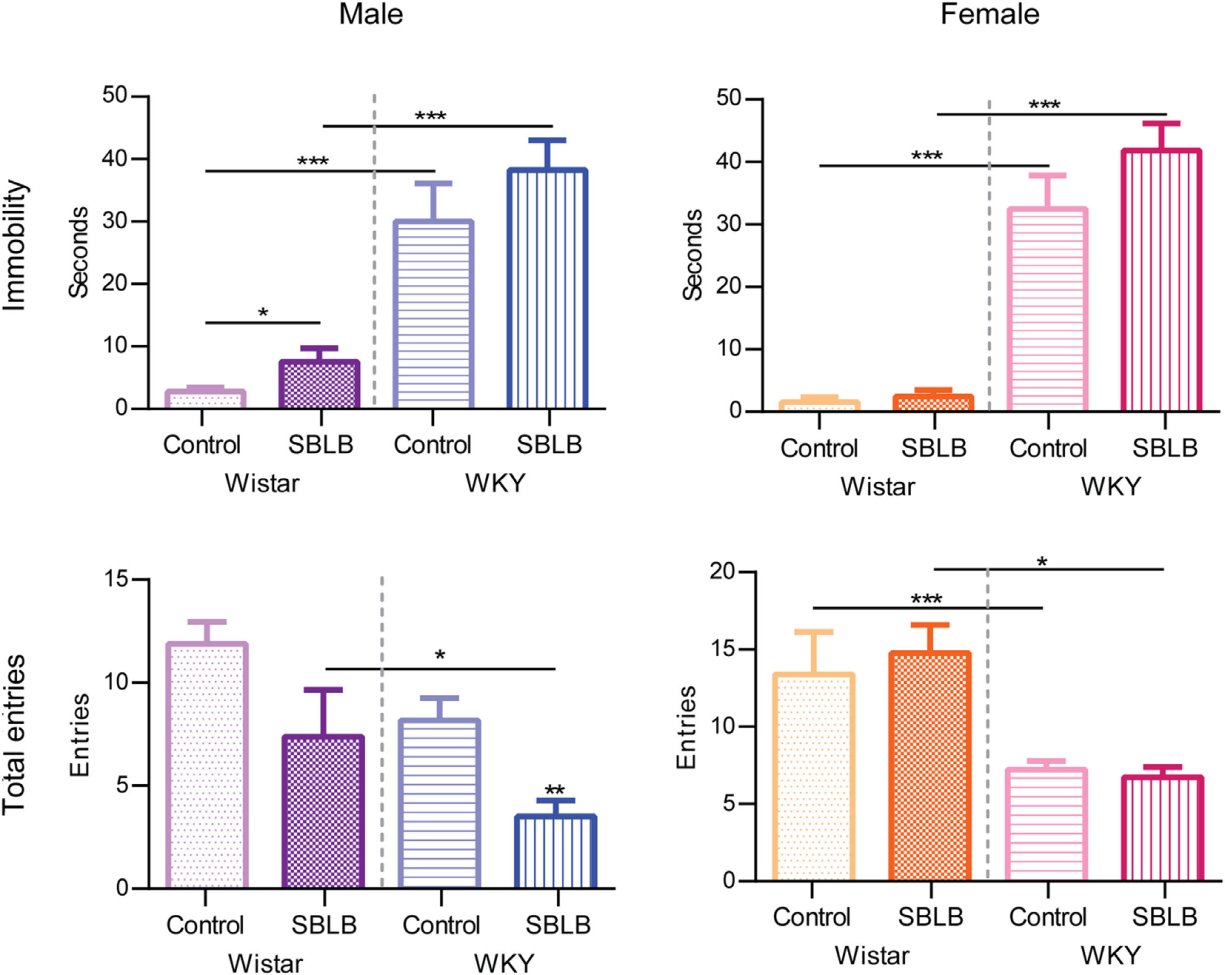
**Total entries and immobility time on the plus-maze test divided by sex and strain**. Two-way ANOVA with Sidak’s multiple comparison test (*P < 0.05, **P < 0.01, ***P < 0.001).

Figure 8 shows the immobility behavior (freezing behavior) and the total number of entries in the PMT. When compared to W rats, male and female WKY rats showed significantly higher immobility behavior in both control and SBLB groups. In W female rats, the SBLB-inducing protocol increased this behavior in comparison to their control group. No differences were observed in male W and female W and WKY rats.

### Serotonin and NA Concentrations on Reward Circuit Related Areas

**Figure 9:**
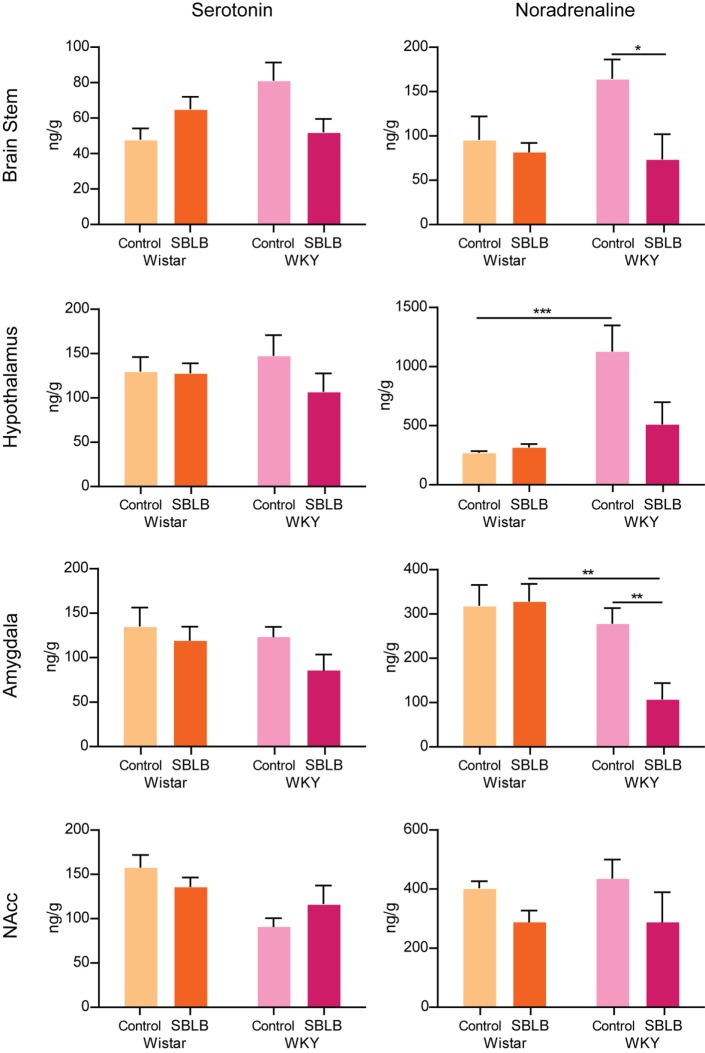
**Tissular concentration of serotonin and noradrenaline in the brain stem, hypothalamus, amygdala, and nucleus accumbens of female Wistar and Wistar-Kyoto (WKY) rats minutes after the ingestion of the 12 test period of the sugar binging-like behavior protocol**. Two-way ANOVA with Sidak’s multiple comparison test. *N* = 6–8 animals per group (*P < 0.05, **P < 0.01, *P* < 0.001).

In the brain stem, we found no differences between the concentrations of 5-HT neither between strains nor treatments, these same results were replicated in the other three brain areas studied (Figure 9).

In the case of NA, between WKY and W female control animals, differences were found only in the hypothalamus. The SBLB-inducing protocol produced no changes on any of the brain areas studied on the W rats. On the hand, SBLB-inducing protocol diminished NA concentrations in the brain stem, amygdala, and hypothalamus. No differences were observed in the NAcc.

### Analysis of the Effect of Fluoxetine on SBLB

**Figure 10:**
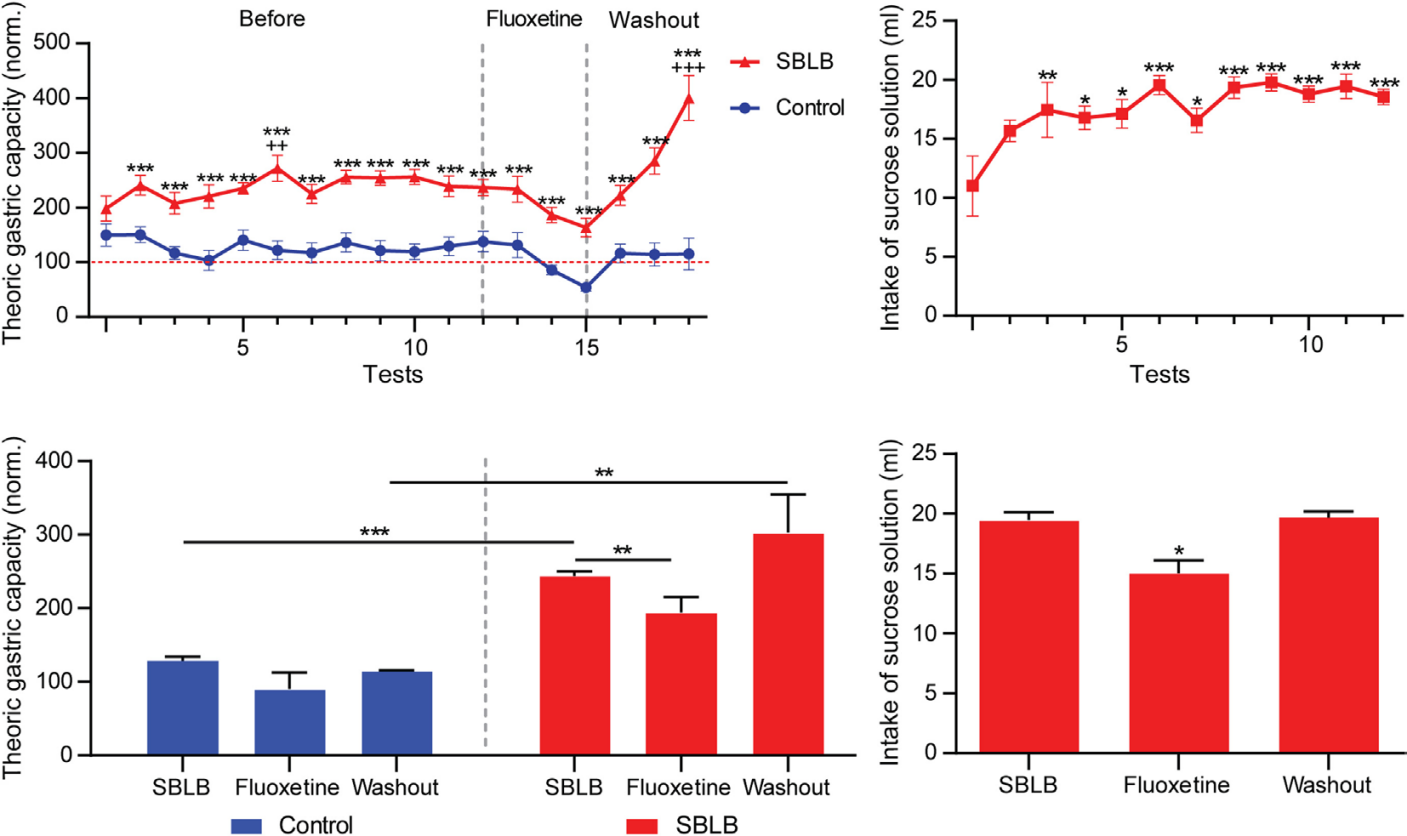
**The sugar binging-like behavior (SBLB)-inducing protocol fulfils all requirements to be considered a valid SBLB-inducing protocol**. Upper left panel: Volume of food consumed during test periods compared to the animal’s theoric gastric capacity during the full duration of the SBLB-inducing protocol (12 tests), during the administration of fluoxetine (3 tests) and during the washout period (3 tests). Two-way ANOVA with Sidak’s multiple comparison test (***P < 0.001), one-way ANOVA for repeated measures with Dunnett multiple comparison test (^++^*P* < 0.01, ^+++^*P* < 0.001). Lower left panel: Mean% of the theoric gastric capacity used during the last three tests of the SBLB protocol, during the administration of fluoxetine (10 mg/kg IP), and during the washout period. Two-way ANOVA with Sidak’s multiple comparison test (***P < 0.001, **P < 0.02, *P < 0.5). Upper right panel: Milliliters of sucrose solution consumed during test periods before, during, and after the administration of fluoxetine 10 mg/kg. One-way ANOVA with Dunnett multiple comparison test (*P < 0.01, **P < 0.001, ***P < 0.001). Lower right panel: Mean sucrose solution consumption during the last three test periods of the SBLB protocol, during the administration of fluoxetine and during the washout period. One-way ANOVA with Sidak’s multiple comparison test (*P < 0.05).

Figure 10 shows the results of the SBLB-inducing protocol. In the upper left panel, we can observe that from the second exposition to palatable food onward, animals consumed a significantly higher volume of food vs. the control during the 2 h test period. The upper right panel shows the total amount of milliliters of sucrose solution consumed during test periods. Sucrose solution ingestion had a steady increase that became significant in comparison to the first exposition from the third test period onward.

The lower left panel shows the effect of the subchronic fluoxetine (10 mg/kg i.p.) administration. The drug diminished the volume of food consumed during test periods in both control and SBLB groups, during the washout period the control animal returned to their normal volume of food consumed while in the SBLB animals this parameter significantly increases.

Finally, the lower right panel shows that sucrose solution consumption during the administration of fluoxetine also decreased, returning to normal levels on washout.

## Discussion

### Sugar Binging-Like Behavior Inducing Protocol

The two most important characteristics that an animal model of binge eating should comply with have been established: first, animals should have a significantly higher caloric intake in a small period (at least two times the amount ingested by the control group). Second, this binge eating episodes should be repeated several times and could be either driven by a stressor or intermittent access periods to a highly palatable food (10). The SBLB-inducing protocol used in the present study fulfills both of these characteristics. It shows a consistent SBLB that was observed as soon as the third day of sugar exposure.

In humans, one of the characteristics of a binge eating episode is to eat until feeling uncomfortably full, which is associated to an excessive distention of the stomach (1). To our knowledge, the use of the comparison between the volumes of food eaten during the binge eating episode with the animal’s gastric capacity (as a manner to evaluate the distention of the stomach) has not been used before. There is no information describing the conservation of the size of the stomach between rat strains, neither about the relation between weight and stomach size in WKY rats. The formula used in this study was developed using Sprague-Dawley (SD) rats and related the size of the stomach with body weight (41), and its results matched the reported stomach size in female W rats (43). Because these two rat strains are quite similar to WKY rats, we considered that using this formula to predict the size of the stomach of this rat strain could be a fair enough approximation. Using this tool, which measures the distention of the stomach during the binge eating episodes, can mimic the characteristic seen in humans.

One of the antidepressants that have shown to be effective in the treatment of BED is fluoxetine (34). This compound decreases the frequency of binge eating episodes and its severity and has been used before in animal models to inhibit binge eating-like behavior (36, 44–47). Therefore, we decided to evaluate if the administration of fluoxetine was able to inhibit the SBLB in our experimental conditions.

In W female rats, fluoxetine was able to significantly diminish the amount of sucrose solution ingested during test periods in the SBLB group. In the control group, the volume of food was also decreased. Anorexic effects of fluoxetine have been reported before only on depressed or BED patients, but not on healthy controls, future studies should analyze this specific effect (48, 49).

In both control and SBLB groups, the anorexic effect observed was lost during the washout period. However, in the SBLB group, caloric ingestion during test periods was magnified. Because fluoxetine is a selective serotonin reuptake inhibitor and in patients with ED, a dysregulation of this neurotransmitter pathway has been reported, and relapsing is common. These results suggest a possible 5-HT mediated relapse effect. Future studies should analyze this hypothesis (26, 50, 51).

### Differences in Response to the SBLB-Inducing Protocol

The second part of this study analyzes the differences between sex and strain on the caloric intake during the SBLB-inducing protocol. Because no differences were observed in the sucrose preference test, the differences that were seen in this study are not due to a different sucrose preference neither between strains nor sexes.

Caloric intake in animals is defined by its basal metabolism plus the energy used in their daily activities (52). As seen in Figure 4, the basal caloric intake per gram of weight of the WKY rat strain female and male was significantly minor than the ones of the W rat strain. It is known that WKY female rats have leaner phenotypes than other rat strains as well as different metabolic responses to exercise (53). Also, WKY female rats have different sedentary metabolic rates (MR) and respiratory quotient (RQ: CO_2_ eliminated/O_2_ consumed) than W rats (MR: 8.7 vs. 8.5 and RQ: 0.84 vs. 0.88), this suggests that the molecules used to obtain energy on a daily basis also change (53). Altogether, these parameters might influence caloric intake and could explain the differences observed in this study. More studies are needed to further analyze these differences.

Moreover, female in general had a better response to the SBLB-inducing protocol. Especially, the WKY female rats developed a more stable and significant binge eating-like behavior in comparison to their W counterparts, consuming more calories during binge episodes (Figure 2). These rats were not able to compensate this high caloric intake (Figure 3) and consume more sucrose solution than any other of the groups, greatly exceeding their gastric capacity (Figure 5). This result suggests that sex and strain differences can play a role in the development of SBLB.

In relation to sex differences, in humans, the prevalence of binge eating and anxiety-related behaviors is higher in women than in men (4, 5). Reports show that sexual hormones play a major role in the control of the homeostatic part of feeding behavior (49, 54). An example of this control is the one mediated by the 17β-estradiol over the hypothalamic expression of neuropeptide Y and α-melatonin stimulating hormone, molecules that play a role in the control of feeding behavior (55, 56).

It is also known that DA release on the NAcc plays a major role in reward and addiction behavior (57, 58). Continuous dopamine release in the NAcc produced by recurrent episodes of binge eating has been reported and can also produce a sensitization to its response (24, 59, 60). There are also reports that estradiol can further sensitized dopamine responses in NAcc (61), suggesting that probably estradiol could play a role inducing SBLB behavior in female rats. More studies are needed to establish a stronger connection between this phenomenon.

In relation to strain differences, WKY male and female rats had a better response to the SBLB-inducing protocol than their W counterpart. First, they develop binge eating episodes from the first day of the SBLB-inducing protocol, while W rats responded until the third test day. Also, they were not able to compensate high caloric intake during no test days in comparison to the W rats.

Differences between strain vulnerability to develop binge eating-like behavior have been reported using a comparison of W rats vs. SD rats. Similar to this study, the W rats were less vulnerable to develop the behavior than SD female rats (62). Therefore, we could consider that W rats might have a genetic or phenotypic characteristic that protects them from developing binge. This singularity should be further analyzed and can give us valuable data in the future.

This is the first time WKY rats have been tested to measure their performance as a response to an SBLB-inducing protocol and due to their vulnerability to develop anxiety-like behavior, a characteristic also seen in patients with ED, we believe this rat strain better mimic the psychiatric disorder seen in humans. Due to this result, we believe female WKY rats could be considered as a prone to develop SBLB animal model. Furthermore, studies have shown that differences found in the WKY rats vs. other rat strains cause changes in the rewarding properties of cocaine and alcohol, as well as in their seeking behavior (28). SBLB has also been linked to addiction. Thus, this characteristic also supports the idea of the WKY being a good model to mimic SBLB.

Finally, the high caloric intake seen in the animals was not sufficient to produce changes in body weight, characteristic that our model shares with others animal models reported in the literature (18, 46). This could be explained due to the restriction periods observed after test sessions. It is important to note that although a high comorbidity between being overweight or obese and BED has been reported, this characteristic is not used as one of the diagnostic criteria for BED (1, 8, 9).

### Anxiety-Like Behavior

Control WKY showed higher levels of immobility behavior that has been related to anxiety, in comparison to W rats. W and WKY rats have different responses to stimuli known to cause anxiety and depression-like behavior being the most observed difference the higher immobility and freezing behavior of the WKY rats (28, 63–65). They also show and enhanced startle response and higher prepulse inhibition, which can be associated with a hypervigilant state that might be involved in this tendency (66). These animals also have a reduced arousal and behavioral responsivity, which may be related to a deficient noradrenergic reactivity, suggesting an altered ability to cope with stressful situations resulting in higher than normal neuroendocrine responses (67).

On the other hand, the SBLB-inducing protocol in the absence of sucrose solution produced an increase in the anxiety-like behavior regardless of the sex or the strain studied. This behavior has been related to a withdrawal-like syndrome (25). In humans, BEDs overlap with substance abuse disorders, genetically and behaviorally, and patients with binge eating tend to show withdrawal syndromes when access to palatable food is restricted (23, 31, 33, 68). These data further support the idea that our sugar-binging inducing protocol shares characteristics with BED in humans.

### NA and Serotonin Concentrations

Various neurochemical analyses have reported that WKY shows differences in the basal levels of DA, NA, and 5-HT in brain areas that have been related to depression and drug addiction (28). Also, the WKY rats, in comparison to SD rats, show a different density of noradrenaline transporters and serotonin transporters (5-HT) in various brain regions (69).

We found that the basal concentrations of NA in WKY control rats, when comparing with W rats, were significantly higher in the hypothalamus, brain area related to the regulation of stress and anxiety. This difference further supports the proposal that WKY can be considered as a genetic animal model of anxiety and depression (21, 22).

On the other hand, due to the exposure to the SBLB protocol concentrations of NA on the brain stem, hypothalamus and amygdala of WKY female rats decrease (being this reduction significant in the brain stem and the amygdala). These results suggest that the reward properties of sucrose solution could be affecting the noradrenergic transmission and might be related to the greater sensibility of these rats to develop SBLB.

## Conclusion

From our findings we suggest, that the WKY female rats can be considered as a good SBLB animal model, that developed strong binge eating episodes and increased its anxiety-like behavior when no palatable food is available. Also, this strain naturally has an increased predisposition to develop anxiety and depression-like behavior as well as difficulty coping with stressful situations, characteristic shared with patients suffering from ED.

Finally, the SBLB-inducing protocol produced changes in the NA concentrations, but no 5-HT concentration in brain areas related to the regulation of anxiety and feeding behavior, in WKY female rats, but not in W female rats. These changes are an important difference between strains and could be related to their susceptibility to develop SBLB phenotype.

This study was not without limitations. The impact of the estrous cycle of the animals over the results of this research cannot be ruled out, but using at least 10 animals per group and having experiments lasting more than four full cycles, the effects of this cycle would be probably averaged out. To our knowledge, there is no information regarding differences on the estrous cycle between strains. Furthermore, in 2016, a meta-analysis made on different traits on behavioral neuroscience data has concluded that even when female rats are used in neuroscience experiments without regard to the estrous cycle stage, their data are not more variable than those of male rats (70).

More studies to further analyze the differences between sex and strain in the development and maintenance of SBLB using stressor-free protocols, and comparing them with the ones using privation to induce the behavior, are needed to explore more deeply their differences and their possible outcomes toward the understanding of the neurobiology of binge eating.

## Ethics Statement

This study was carried out in accordance with the recommendations of the Mexican Official Norm (NOM-062-ZOO-1999) for the use and care of laboratory animals. The protocol was approved by CINVESTAV’s ethics committee (Protocol 0179-16).

## Author Contributions

HP-Q did the experimental work, statistical analysis, wrote the manuscript, participated in the conception and design of the experiments, and helped during the revision process. VO-O participated in the conception and design of the experiments and assisted in the review process. CL-R participated during the conception and design of the experiments, during the review process and approved the final version of this manuscript.

## Conflict of Interest Statement

The authors declare that the research was conducted in the absence of any commercial or financial relationships that could be construed as a potential conflict of interest.
